# Predicting incidence of long-term care insurance certification in Japan with the Kihon Checklist for frailty screening tool: analysis of local government survey data

**DOI:** 10.1186/s12877-020-01968-z

**Published:** 2021-01-07

**Authors:** Kumiko Ito, Hisashi Kawai, Harukazu Tsuruta, Shuichi Obuchi

**Affiliations:** Tokyo Metropolitan Institute of Gerontology, 35-2 Sakae-cho, Itabashi-Ku, Tokyo, 173-0015 Japan

**Keywords:** Older persons, Kihon checklist, Long-term care insurance certification, Risk prediction, Screening

## Abstract

**Background:**

Predicting incidence of long-term care insurance (LTCI) certification in the short term is of increasing importance in Japan. The present study examined whether the Kihon Checklist (KCL) can be used to predict incidence of LTCI certification (care level 1 or higher) in the short term among older Japanese persons.

**Methods:**

In 2015, the local government in Tokyo, Japan, distributed the KCL to all individuals older than 65 years who had not been certified as having a disability or who had already been certified as requiring support level 1–2 according to LTCI system. We also collected LTCI certification data within the 3 months after collecting the KCL data. The data of 17,785 respondents were analyzed. First, we selected KCL items strongly associated with incidence of LTCI certification, using stepwise forward-selection multiple logistic regression. Second, we conducted receiver operating characteristic (ROC) analyses for three conditions (1: Selected KCL items, 2: The main 20 KCL items (nos. 1–20), 3: All 25 KCL items). Third, we estimated specificity and sensitivity for each condition.

**Results:**

During a 3-month follow-up, 81 (0.5%) individuals required new LTCI certification. Eight KCL items were selected by multiple logistic regression as predictive of certification. The area under the ROC curve in the three conditions was 0.92–0.93, and specificity and sensitivity for all conditions were greater than 80%.

**Conclusions:**

Three KCL conditions predicted short-term incidence of LTCI certification. This suggests that KCL items may be used to screen for the risk of incident LTCI certification.

## Background

The long-term care insurance (LTCI) system was introduced in Japan in the year 2000 to support people with disabilities via social security expenses [1]. The number of LTCI certifications in Japan is estimated to reach 9.88 million (8.9% of the total population) by 2040, a figure that is approximately double that of 2010 [2]. Increasing social security expenses represents a serious future issue [3].

The LTCI system in Japan provides people with services according to certification as one of seven levels (support levels 1–2 and care levels 1–5) depending on their disease condition or functional ability. LTCI care levels require more care than LTCI support levels, and the higher the level, the more care required. LTCI certification levels are defined for example as follows Support level 1: “limited in instrumental activities of daily living but independent in basic activities of daily living”; care level 2: “requiring assistance in at least one basic task of activities of daily living”; care level 5: “requiring care in all tasks of activities of daily living.” People with support level 1–2 can receive preventive services, as may people who are not certified as having a disability [1]. To assess whether a person is eligible for preventive services or LTCI services, use of the Kihon Checklist (KCL) has been recommended by the Ministry of Health, Labour and Welfare, of Japan [4]. The KCL is a screening tool developed in Japan for use with older people with frailty [5]. It is used by local governments and in community consultations to screen for persons eligible for long-term care prevention programs and to assess the effectiveness of interventions. The KCL was automatically sent to all individuals older than 65 years on an annual basis up until 2014, but is now administered at the discretion of each local government. The KCL consists of 25 items; it requires approximately 15 min for an older person to answer all items [6].

In Japan, LTCI certifications are determined at the Municipal Certification Committee, based on the assessment of the degree of functional disability using a questionnaire developed by the Ministry of Health, Labour and Welfare and reference to the “Doctor’s Opinion Paper” prepared by the attending physician [1]. The “Doctor’s Opinion Paper” contains the opinion of the primary care doctor on the applicant’s chronic condition in daily life that cannot be determined by the questionnaire. There is a cost of 10,000 to 20,000 Yen (100 to 200 US dollars) per applicant associated with determining LTCI certification at the Municipal Certification Committee [7]. However, if preventive services were provided to applicants who are not assessed as requiring care level 1 or higher based on KCL screening, the costs of unnecessary certification would reduce. Preventive services are programs for individuals who are not certified as having a disability or have LTCI support level 1–2, and are thus available without LTCI certification. One preventive service (service “C”) aims to gain intensive improvement in participants’ activities of daily living, instrumental activities of daily living, and physical fitness over a short term of 3 to 6 months; the service program is reviewed a minimum of 3 months after onset [4]. Therefore, to reduce social security expenses, it is necessary to use the KCL to determine persons who will likely change their care needs to care level 1 or higher in a short term of 3 months.

Previous studies have shown that each item and the seven domains of the KCL are associated with incidence of LTCI certification [8–13]. One study reported that each domain of the KCL was useful for predicting the occurrence of LTCI certification (support level 1 or higher) in the subsequent year; the main 20 items of the KCL (nos. 1–20) had the greatest predictive ability [9]. Another study reported that the nutrition domain, memory domain, and mood domain of the KCL were associated with 2-year incidence of LTCI certification (care level 2 or higher) [10].

However, these studies examined the association between the KCL and incidence of LTCI certification over more than a 1-year period; to the best of our knowledge, the ability of the KCL to predict incidence of LTCI certification in the short term has not been reported. Moreover, it would be useful to be able to predict incidence of LTCI certification using a subset of KCL items focused on practical situations.

Accordingly, the present study examined whether the KCL can be used to predict incidence of LTCI certification (care level 1 or higher) in the short term. To do so, we compared the utility of different KCL items for predicting incidence of LTCI certification during a 3-month period, among the different item sets, using a large dataset collected by a local government in Japan.

## Methods

### Participants

We used KCL data that were collected by the local government in Tokyo, Japan, in May 2015 to screen for frail older persons. The KCL was distributed by mail to 26,630 individuals older than 65 years who were not certified as with a disability or with LTCI support level 1–2; the data of 17,785 individuals (66.8%) who provided responses were analyzed. The ethics committee of the Tokyo Metropolitan Institute of Gerontology approved the present study. We explained in writing to the local government the purpose of the present study and how the results would be disseminated, and we obtained written consent to use the data.

### KCL at baseline

The KCL consists of 25 self-report (yes/no) questions that assess seven domains: instrumental activities of daily living, physical strength, nutrition, oral function, isolation, memory, and mood [5]. The main 20 items of the KCL (nos. 1–20), which are often used as the overall score of frailty, include six domains and exclude a mood domain [8]. Responses are summed to obtain a KCL score for each participant. A higher KCL score indicates a higher risk of frailty.

### Follow-up (incidence of LTCI certification)

Combined anonymized data on both the KCL and incidences of LTCI certification in the 3 months after collecting the KCL data were retrieved from the local government. Incidence of LTCI certification was defined as care level 1 or higher.

### Statistical analysis

First, we used stepwise forward-selection logistic regression analysis, adjusting for sex and age, and using all 25 items of the KCL as explanatory variables to determine KCL items that were strongly associated with incidence of LTCI certification. We conducted receiver operating characteristic (ROC) analyses to calculate the area under the curve (AUC) and 95% confidence intervals for incidence of LTCI certification for following three conditions: i) KCL items selected based on the regression analysis described above, ii) the main 20 items of the KCL (nos. 1–20) which had better predictive utility based on previous study [9], and iii) all 25 items of the KCL. We additionally conducted a five-fold cross validation test for the selected items of the KCL. We created five data sets by randomly splitting all of the data into a training set and a test set with the ratio of 70–30%, and estimated the accuracy of predicting incidence of LTCI certification. Third, we estimated the specificity and sensitivity for incidence of LTCI certification in each condition, based on the Youden Index value [14, 15]. Specificity indicates the rate of individuals who are assessed as not requiring LTCI certification when they are not certified, and sensitivity indicates the rate of individuals who are assessed as requiring it when they are certified. All data were analyzed using SPSS Statistics 23.0 (SPSS Inc., Chicago, IL, USA). All statistical tests were two-sided, and differences at *P* < 0.05 were accepted as significant.

## Results

### Population characteristics

The study population comprised 7827 men (44.0%) and 9958 women (56.0%), with a median age of 74 years (range: 65–107 years). During the 3-month follow-up, 81 persons (0.5%) required new LTCI certification.

### Selected KCL items

Table 1 shows the eight KCL items that were selected using the regression, and sex- and age-adjusted odds ratios (ORs) and confidence intervals (CIs) of incidence of LTCI certification for each item. The selected eight KCL items included three items from the instrumental activities of daily living domain, one item from the physical strength domain, one item from the nutrition domain, one item from the memory domain, and two items from the mood domain.

**Table 1:** Association between the selected eight Kihon checklist items and incidence of long-term care insurance certification

	Incidence of long-term care insurance certification			ORs (95% CIs) ^a^
	n	Case	(%)	
1. Do you go out by bus or train by yourself?				
Yes	16,593	30	(0.2)	1.00 (reference) ^b^
No (positive)	1192	51	(4.3)	3.41 (1.96–5.92)
2. Do you go shopping to buy daily necessities by yourself?				
Yes	16,764	37	(0.2)	–
No (positive)	1021	44	(4.3)	–
3. Do you manage your own deposits and savings at the bank?				
Yes	16,280	37	(0.2)	1.00 (reference)
No (positive)	1505	44	(2.9)	2.54 (1.50–4.28)
4. Do you sometimes visit your friends?				
Yes	12,537	10	(0.1)	1.00 (reference)
No (positive)	5248	71	(1.4)	3.59 (1.74–7.44)
5. Do you turn to your family or friends for advice?				
Yes	15,194	31	(0.2)	–
No (positive)	2591	50	(1.9)	–
6. Do you normally climb stairs without using handrail or wall for support?				
Yes	12,348	13	(0.1)	1.00 (reference)
No (positive)	5437	68	(1.3)	3.01 (1.58–5.72)
7. Do you normally stand up from a chair without any aids?				
Yes	15,370	30	(0.2)	–
No (positive)	2415	51	(2.1)	–
8. Do you normally walk continuously for 15 min?				
Yes	16,350	35	(0.2)	–
No (positive)	1435	46	(3.2)	–
9. Have you experienced a fall in the past year?				
No	14,205	35	(0.2)	–
Yes (positive)	3580	46	(1.3)	–
10. Do you have a fear of falling while walking?				
No	10,893	14	(0.1)	–
Yes (positive)	6892	67	(1.0)	–
11. Have you lost 2 kg or more in the past 6 months?				
No	14,944	45	(0.3)	1.00 (reference)
Yes (positive)	2841	36	(1.3)	1.71 (1.06–2.76)
12. If BMI is less than 18.5, this item is scored.				
No	15,872	57	(0.4)	–
Yes (positive)	1913	24	(1.3)	–
13. Do you have any difficulties eating tough foods compared to 6 months ago?				
No	13,805	39	(0.3)	–
Yes (positive)	3980	42	(1.1)	–
14. Have you choked on your tea or soup recently?				
No	13,965	50	(0.4)	–
Yes (positive)	3820	31	(0.8)	–
15. Do you often experience having a dry mouth?				
No	13,349	46	(0.3)	–
Yes (positive)	4436	35	(0.8)	–
16. Do you go out at least once a week?				
Yes	16,686	53	(0.3)	–
No (positive)	1099	28	(2.5)	–
17. Do you go out less frequently compared to last year?				
No	13,959	25	(0.2)	–
Yes (positive)	3826	56	(1.5)	–
18. Do your family or your friends point out your memory loss?				
No	16,113	47	(0.3)	–
Yes (positive)	1672	34	(2.0)	–
19. Do you make a call by looking up phone numbers?				
Yes	15,967	43	(0.3)	1.00 (reference)
No (positive)	1818	38	(2.1)	2.10 (1.29–3.42)
20. Do you find yourself not knowing today’s date?				
No	14,766	41	(0.3)	–
Yes (positive)	3019	40	(1.3)	–
21. In the last 2 weeks have you felt a lack of fulfilment in your daily life?				
No	14,762	32	(0.2)	–
Yes (positive)	3023	49	(1.6)	–
22. In the last 2 weeks have you felt a lack of joy when doing the things you used to enjoy?				
No	15,848	29	(0.2)	1.00 (reference)
Yes (positive)	1937	52	(2.7)	3.21 (1.88–5.48)
23. In the last 2 weeks have you felt difficulty in doing what you could do easily before?				
No	13,134	21	(0.2)	–
Yes (positive)	4651	60	(1.3)	–
24. In the last 2 weeks have you felt helpless?				
No	14,356	26	(0.2)	1.00 (reference)
Yes (positive)	3429	55	(1.6)	1.90 (1.11–3.26)
25. In the last 2 weeks have you felt tired without a reason?				
No	13,063	31	(0.2)	–
Yes (positive)	4722	50	(1.1)	–

^a^Odds ratio (95% confidence interval)

^b^Adjusted for sex and age

### ROC analysis

**Fig. 1:**
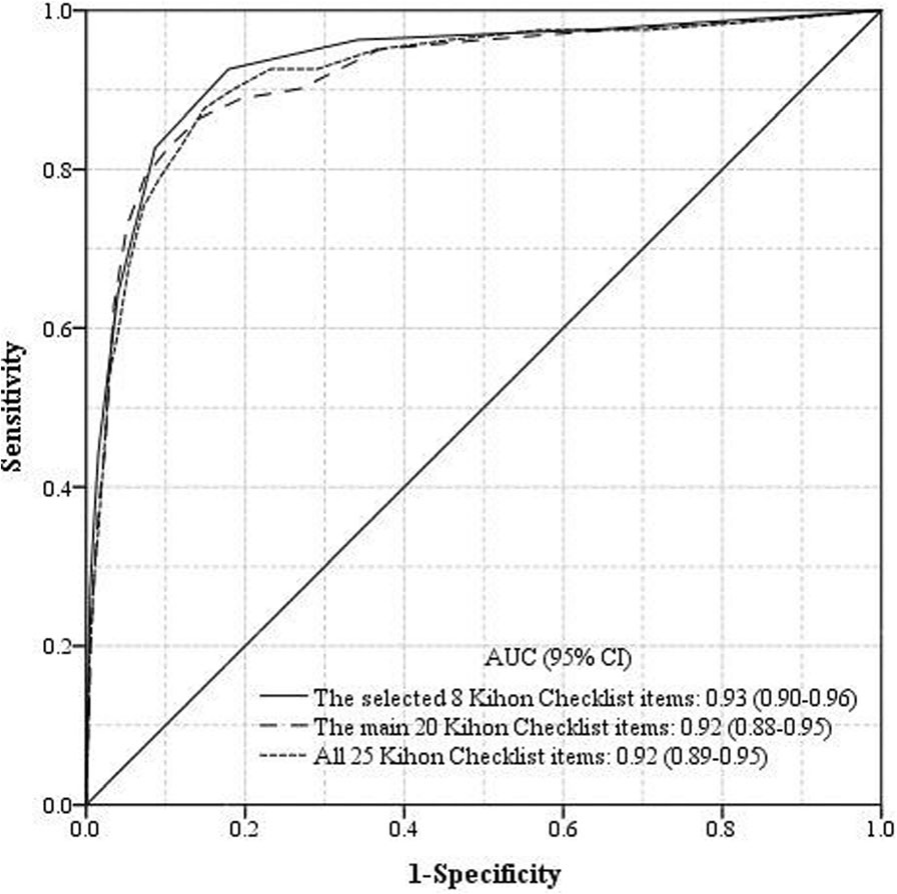
Area under the receiver operating characteristic curve in the three Kihon Checklist conditions

The AUC of the selected eight KCL items was 0.93 (95% CI 0.90–0.96), which was the highest of the three conditions (Fig. 1). The AUCs of the main 20 items and all 25 items were 0.92 (95% CI 0.88–0.95) and 0.92 (95% CI 0.89–0.95), respectively. The five-fold cross validation test for the selected eight items indicated that the average AUC of the five training sets was 0.94, and that of the five test sets was 0.92.

### Specificity and sensitivity

The optimal cut-off points determined by the Youden Index were ≥ 3/8 for the selected eight items (specificity 82.1%, sensitivity 92.6%), ≥8/20 for the main 20 items (specificity 89.7%, sensitivity 82.7%), and ≥ 9/25 for all 25 items (specificity 85.1%, sensitivity 87.7%; Table 2).

**Table 2:** Specificity and sensitivity according to the cut-off points in the three Kihon Checklist conditions

	Incidence of long-term care insurance certification			Specificity	Sensitivity
	n	Case	(%)		
The selected eight Kihon Checklist items					
3 points ^a^				82.1%	92.6%
< 3 points	14,536	6	(0.0)		
≥ 3 points	3168	75	(2.3)		
4 points ^a^				91.3%	82.7%
< 4 points	16,167	14	(0.1)		
≥ 4 points	1537	67	(4.2)		
The main 20 Kihon Checklist items					
8 points ^a^				89.7%	82.7%
< 8 points	15,880	14	(0.1)		
≥ 8 points	1824	67	(3.5)		
All 25 Kihon Checklist items					
9 points ^a^				85.1%	87.7%
< 9 points	15,074	10	(0.1)		
≥ 9 points	2630	71	(2.6)		

^a^Cut-off point

## Discussion

In the present study, we investigated that whether the KCL can be used to predict incidence of LTCI certification (care level 1 or higher) in the short term. Eight KCL items that were strongly associated with LTCI certification included three items from the instrumental activities of daily living domain, one item from the physical strength domain, one item from the nutrition domain, one item from the memory domain, and two items from the mood domain. The AUC of these eight items with respect to incidence of LTCI certification was 0.93, which was as high as that of the main 20 items and all 25 items. This result suggests that the eight items sufficiently predicted short-term incidence of LTCI certification.

There was likely no significant difference in the age distribution of the participants in the present study and overall older population in Japan because we used large-scale data from complete enumeration obtained by the local government [16]. Moreover, the proportion of participants with LTCI certification (support level 1–2) at baseline was comparable to that of the whole Japanese population [16, 17]; thus, the data in the present study were representative of community-dwelling older persons in Japan. Tomata [9] reported that the 2.2% of individuals required new LTCI certification (care level 1 or higher) during a one-year follow-up, versus 0.5% during a three-month follow-up in the present study; the latter value might have been affected by the shorter follow-up period, however, we believe that the accuracy of the statistical analysis performed on the large-scale sample of 17,785 people is sufficient, and that the findings obtained can be generalized.

Tomata reported that the items of the KCL that were associated with 1-year incidence of LTCI certification (support level 1 or higher), through forced-entry logistic regression analysis, were two items from the instrumental activities of daily living domain, two items from the physical strength domain, one item from the nutrition domain, one item from the isolation domain, and three items from the cognitive function domain [9]. Of these items, item 4 (“Do you sometimes visit your friends?”), of the domain of instrumental activities of daily living, and item 19 (“Do you make a call by looking up phone numbers?”), of the cognitive function domain, were also selected in our study. In addition to these items, items from the mood domain were selected in the present study. Fukutomi reported no association between the mood domain and two-year incidence of LTCI certification (support level 1 or higher) [11]. However, Hamazaki reported a significant association between the mood domain and two-year incidence of LTCI certification (care level 2 or higher) [10]. Therefore, items of the mood domain might be associated with short-term and more severe need for LTCI certification.

The utility of KCL items for predicting incidence of LTCI certification in the present study was notably greater than that reported in previous studies: The AUC in ROC analysis was 0.62–0.83 in Tomata et al. [9] and 0.78 in Tsuji et al. [13] The present study focused on the need for LTCI certification at care level 1 or higher over a shorter follow-up period than that in previous studies; the results suggested that the KCL was useful for predicting incidence of LTCI certification in the short term. Moreover, we found that the eight selected items were as efficacious for predicting incidence of LTCI certification as were the main 20 items and all 25 items. This result was also confirmed by a five-fold cross validation test.

The specificity and sensitivity for incidence of LTCI certification based on the selection criteria of the LTCI for people at high risk were 57.8 and 73.5%, respectively, according to national research in Japan, and 63.4 and 78.1%, respectively, in Tomata et al. [9] Moreover, specificity and sensitivity were 73.1 and 70.5%, respectively in a previous study that used part of the KCL [13]. Specificity and sensitivity in the present study were both more than 80%, values higher than those reported in previous studies.

In the present study, sensitivity using the eight selected items was highest at 92.6%, using a cut-off point of ≥3/8 items; however, specificity was highest at 91.3%, using a cut-off point of ≥4/8 items. These results suggest that it might be useful to change the cut-off point of the eight selected items according to various situations. The ≥3/8 cut-off point might be recommended if fewer false negatives are required during primary screening, while the ≥4/8 cut-off point might be recommended if fewer false positives are required during medical examinations.

Using the results of the present study, we simulated the reduction in social security expenses when the local government determined no LTCI certification at Municipal Certification Committees for service applicants who were assessed as not having a disability or as having LTCI support level 1–2, based on the KCL. In the 2015 national report, there were one million people targeted for them. Thus, this would be expected to reduce social security expenses by 10 billion Yen (100 million US dollars) per year, given a cost of 10,000 Yen (100 US dollars) per person to determine LTCI certification at the Municipal Certification Committee [7, 18]. It is recommended that local governments evaluate applicants for LTCI services using the eight items of the KCL to determine whether LTCI services or preventive services are suitable. If by using the eight items, older adults are deemed unlikely to be certified as requiring care level 1 or higher after 3-months, they may use preventive services. Globally, governments seeking to introduce LTCI might be able to reduce social security expenses by using the eight items to conduct a primary screening to assess those requiring LTCI certification before determining the service applicant’s LTCI certification.

The present study had several limitations. We used data that were collected by the local government to screen for older persons with frailty; however, the response rate was 66.8%. Thus, the study population might not have included people at higher risk of incidence of LTCI certification. Because not all participants eligible for LTCI certification actually apply for LTCI certification, the present study might have included detection bias. Since we did not investigate disease and functional ability, how health status affected LTCI certification was unclear.

## Conclusions

In conclusion, the present study showed that the main 20 items and all 25 items could be used to predict incidence of LTCI certification during a subsequent 3-month period with high accuracy. Moreover, eight selected items of the KCL also could be used to make this prediction. Therefore, the items of the KCL that are included could be changed according to various situations when predicting short-term incidence of LTCI certification.

## Supplementary Information

**Additional file 1.**

## Data Availability

The data of this study cannot be released publicly due to ethicolegal restrictions imposed by the Ethics Committee at Tokyo Metropolitan Institute of Gerontology. Datasets generated may be available from the corresponding author on reasonable request, after ethical considerations.

## Abbreviations

LTCI: Long-term care insurance
KCL: Kihon Checklist
ROC: Receiver operating characteristic
AUC: Area under the curve
OR: Odds ratio
CI: Confidence interval
BMI: Body mass index
