# Supplementary material for: Predicting incidence of long-term care insurance certification in Japan with the Kihon Checklist for frailty screening tool: analysis of local government survey data
Source: BMC Geriatr. 2021 Jan 7;21:22. doi: 10.1186/s12877-020-01968-z (PMC7792049; doi:10.1186/s12877-020-01968-z)
Supplement: Supplementary file 1 — Additional file 1. [file 12877_2020_1968_MOESM1_ESM.docx]

Kihon checklist

| Questionnaire | Answer | |
| --- | --- | --- |
| 1. Do you go out by bus or train by yourself? | 0. Yes | 1. No |
| 2. Do you go shopping to buy daily necessities by yourself? | 0. Yes | 1. No |
| 3. Do you manage your own deposits and savings at the bank? | 0. Yes | 1. No |
| 4. Do you sometimes visit your friends? | 0. Yes | 1. No |
| 5. Do you turn to your family or friends for advice? | 0. Yes | 1. No |
| 6. Do you normally climb stairs without using handrail or wall for support? | 0. Yes | 1. No |
| 7. Do you normally stand up from a chair without any aids? | 0. Yes | 1. No |
| 8. Do you normally walk continuously for 15 min? | 0. Yes | 1. No |
| 9. Have you experienced a fall in the past year? | 1. Yes | 0. No |
| 10. Do you have a fear of falling while walking? | 1. Yes | 0. No |
| 11. Have you lost 2 kg or more in the past 6 months? | 1. Yes | 0. No |
| 12. If BMI is less than 18.5, this item is scored. | 1. Yes | 0. No |
| 13. Do you have any difficulties eating tough foods compared to 6 months ago? | 1. Yes | 0. No |
| 14. Have you choked on your tea or soup recently? | 1. Yes | 0. No |
| 15. Do you often experience having a dry mouth? | 1. Yes | 0. No |
| 16. Do you go out at least once a week? | 0. Yes | 1. No |
| 17. Do you go out less frequently compared to last year? | 1. Yes | 0. No |
| 18. Do your family or your friends point out your memory loss? | 1. Yes | 0. No |
| 19. Do you make a call by looking up phone numbers? | 0. Yes | 1. No |
| 20. Do you find yourself not knowing today’s date? | 1. Yes | 0. No |
| 21. In the last 2 weeks have you felt a lack of fulfilment in your daily life? | 1. Yes | 0. No |
| 22. In the last 2 weeks have you felt a lack of joy when doing the things you used to enjoy? | 1. Yes | 0. No |
| 23. In the last 2 weeks have you felt difficulty in doing what you could do easily before? | 1. Yes | 0. No |
| 24. In the last 2 weeks have you felt helpless? | 1. Yes | 0. No |
| 25. In the last 2 weeks have you felt tired without a reason? | 1. Yes | 0. No |

BMI: Body mass index
